# Dynamic Graph Neural Network for Vehicle Trajectory Prediction and Driving Intent Recognition

**DOI:** 10.3390/s26092826

**Published:** 2026-05-01

**Authors:** Shaobo Wu, Yuxuan Wang, Yi Gong

**Affiliations:** College of Information and Communication Engineering, Beijing Information Science and Technology University, Beijing 102206, China; wushaobo@bistu.edu.cn (S.W.); gongyi@bistu.edu.cn (Y.G.)

**Keywords:** vehicle trajectory prediction, driving intent recognition, dynamic graph neural network, transformer, interactive behavior modeling, motion trend intent completion, autonomous driving

## Abstract

To address the limitations of existing vehicle trajectory prediction methods, including insufficient modeling of dynamic inter-vehicle interactions, weak temporal continuity of complex driving intentions such as lane-changing, and high uncertainty in future trajectory prediction, this paper proposes a vehicle trajectory prediction method that integrates Dynamic Graph Neural Networks (DyGNN) with Transformer. Specifically, a time-varying interaction graph is constructed to model the dynamically evolving topological interaction relationships among vehicles, while a Transformer encoder is employed to extract temporal dependency features from historical trajectory sequences. In this way, the joint representation of spatial interaction information and temporal evolution information is achieved, thereby improving the accuracy and continuity of driving intention recognition in complex traffic scenarios. On this basis, driving intention is further introduced into the trajectory prediction process as a prior constraint, which effectively reduces the uncertainty of future trajectory prediction. Comparative experiments on real-world traffic datasets demonstrate that the proposed method maintains low prediction errors across different prediction horizons, showing good effectiveness and robustness.

## 1. Introduction

In highway scenarios, high traffic density and large speed variations give rise to complex and highly dynamic interactions among vehicles. During vehicle intent recognition, particularly under such conditions, considerable uncertainty is often present. If the intentions of surrounding vehicles cannot be identified in a timely and accurate manner, and their future trajectories cannot be reliably predicted, traffic conflicts or even accidents may occur, thereby negatively affecting road efficiency and driving safety [1]. Therefore, research on vehicle intent recognition and trajectory prediction in complex traffic environments is of significant practical importance for improving the perception and decision-making capabilities of autonomous driving systems.

Early studies on driving intent recognition and trajectory prediction primarily relied on traditional probabilistic models and classical machine learning algorithms, such as Hidden Markov Models (HMM) and their variants, including Gaussian Mixture HMM (GMM-HMM) and Support Vector Machines (SVM) [2]. These methods are typically built upon manually designed discrete trajectory features, which makes it difficult to fully capture continuously evolving vehicle motion states and dynamic inter-vehicle interactions. As a result, their adaptability and generalization ability in complex traffic environments remain limited.

With the advancement of data-driven methodologies, neural network-based trajectory prediction models have become a major research focus [3]. Ji Xuewu et al. employed Long Short-Term Memory (LSTM) networks to develop a joint driving intent recognition and trajectory prediction framework, and incorporated a Mixture Density Network (MDN) to model trajectory distributions and improve predictive expressiveness [4]. Xie et al. [5] combined the spatial feature extraction capability of Convolutional Neural Networks (CNNs) with the temporal modeling strength of LSTMs to build a trajectory prediction model for surrounding vehicles. Guo Jinghua et al. [6,7] further introduced a residual bidirectional LSTM architecture, achieving simultaneous improvements in driving behavior recognition and trajectory prediction accuracy. In addition, Yook et al. [8] integrated high-definition map information into deep learning models to strengthen environmental constraint modeling in trajectory prediction. Gao Zhenhai et al. [9] proposed a Multi-Bidirectional LSTM (MB-LSTM) model that incorporates interaction-aware environmental features and demonstrated strong performance in long-horizon trajectory prediction tasks. In addition, Raju et al. [10] proposed an HCNN-based hybrid model that improves prediction accuracy while reducing latency. Liang et al. [11] designed a hybrid CNN–GCN architecture to achieve more accurate trajectory prediction by separately extracting vehicle-specific features and lane-related features. Huang et al. [12] proposed the Spatial-Temporal Graph Attention Network (STGAT), which jointly models spatial interaction relationships among agents and temporal evolution dependencies, thereby effectively improving trajectory prediction performance in complex interactive scenarios and providing an important reference for subsequent graph-based trajectory prediction methods. With the development of attention mechanisms and multi-agent collaborative modeling, S. Mozaffari et al. [13] developed a vehicle trajectory prediction model incorporating a multi-head attention mechanism, which achieves collaborative prediction by integrating multi-vehicle interaction information in traffic scenes. Zhang et al. [14] further proposed a prediction and planning framework integrating a Bayesian–Gaussian mixture model, and experimental results showed that the framework achieved high prediction accuracy over a 5 s prediction horizon.

Although the aforementioned methods have improved trajectory prediction accuracy to a certain degree, most models still rely primarily on sequential modeling and remain limited in their ability to capture complex and time-varying inter-vehicle interactions. In recent years, Dynamic Graph Neural Networks have been increasingly adopted in traffic scene modeling due to their strengths in multi-agent interaction representation. For example, models such as DGInet [15] employ dynamic graph structures to characterize spatiotemporal dependencies among vehicles, thereby improving prediction performance. Meanwhile, the Transformer architecture has demonstrated strong capability in modeling long-range temporal dependencies and global contextual relationships. Methods such as TPNet [16] leverage Transformers to generate multimodal trajectory proposals, significantly enhancing both the diversity and accuracy of prediction outcomes. In addition, generative methods such as diffusion models [17] have gradually been introduced into trajectory prediction tasks to model complex probability distributions in continuous spaces.

Motivated by the above research background, this paper proposes a spatiotemporal interaction modeling framework that integrates a Dynamic Graph Neural Network (DyGNN) with a Transformer architecture for complex highway merge scenarios. First, a dynamic spatiotemporal interaction graph is constructed, and DyGNN is employed to extract structured inter-vehicle interaction features. Subsequently, historical trajectory information is incorporated into a Transformer-based module to infer the intentions of surrounding vehicles. The inferred intent is then introduced into the trajectory prediction stage as a prior constraint to guide future trajectory estimation. This method can simultaneously model vehicle motion characteristics and multi-vehicle interaction relationships, providing effective support for autonomous driving systems to make safe decisions in complex traffic scenarios.

## 2. Scene Description

This study considers a target vehicle, denoted as $S_{0}$. In highway lane-changing and car-following scenarios, the driving intent and trajectory evolution of the target vehicle are determined not only by its own motion state but also by interactions with surrounding vehicles. In general, the most relevant interaction objects for longitudinal following and lateral lane-changing decisions are mainly located in the front and rear regions of the current lane and the adjacent lanes. Therefore, this study selects six neighboring vehicles at representative relative positions as the primary influencing vehicles, namely the left-front vehicle $S_{1}$, left-rear vehicle $S_{2}$, front vehicle $S_{3}$, rear vehicle $S_{4}$, right-front vehicle $S_{5}$, and right-rear vehicle $S_{6}$. This setting can effectively capture the key local interaction relationships in the surrounding traffic environment while avoiding excessive information redundancy and additional model complexity caused by introducing distant vehicles.

Therefore, the state variables of the target vehicle and its interacting surrounding vehicles are adopted as input features for the driving intent recognition and trajectory prediction model. Their historical state information can be formally represented as follows:

(1) $$I\left(t\right)=(S_{0}\left(t\right),S_{1}\left(t\right),S_{2}\left(t\right),S_{3}\left(t\right),S_{4}\left(t\right),S_{5}\left(t\right),S_{6}\left(t\right))$$

(2) $$S_{i}\left(t\right)=\left(x\left(t\right),y\left(t\right),v\left(t\right)\right)$$

(3) $$t=(T-T_{p},\ldots ,T-1,T)$$

(4) $$O\left(t\right)=\left(X\left(t\right),Y\left(t\right)\right),\;t=\left(T+1,T+2,\ldots ,T+T_{F}\right)$$

Here, $S_{i}\left(t\right)$ denotes the state of the vehicle at time *t* at the *i*-th relative position. Specifically, x(t) and y(t) represent the lateral and longitudinal position coordinates at time *t*, respectively, and v(t) denotes the vehicle’s absolute velocity. Equation (3) defines the historical observation time window, where *T* represents the current time step and $T_{P}$ denotes the length of the historical observation horizon. Based on the historical state information, the model is required to predict the trajectory of the target vehicle over a future prediction horizon. In Equation (4), X(t) and Y(t) denote the predicted lateral and longitudinal positions of the vehicle at future time step *t*, respectively, while $T_{F}$ represents the length of the prediction horizon. In real traffic scenarios, certain relative positions around the target vehicle may be unoccupied at specific time steps. To handle this situation, a placeholder encoding strategy is adopted. Specifically, when no vehicle is present at the *i*-th forward relative position at time *t*, the relative distance is treated as infinite, and its state vector is assigned as $S_{i}\left(t\right)=\left[999,\;999,\;0\right]$, Similarly, when no vehicle exists at the *i*-th rear relative position at time *t*, the relative distance is also treated as infinite, and the corresponding state vector is defined as $S_{i}\left(t\right)=\left[-999,\;-999,\;0\right]$.

**Figure 1:**
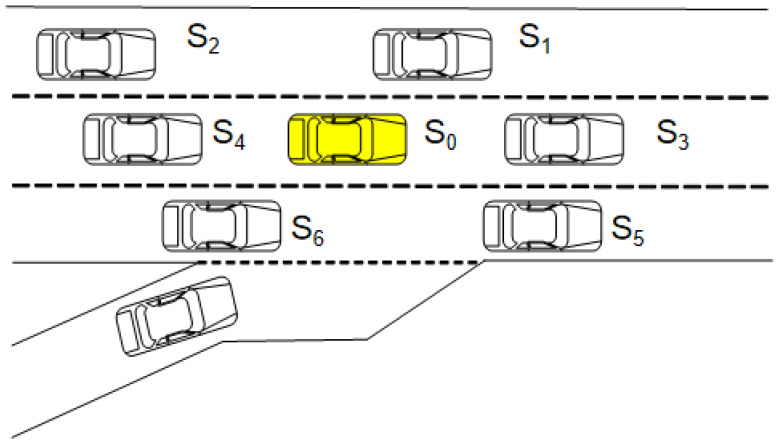
Vehicle Driving Environment Diagram.

As illustrated in Figure 1, inter-vehicle interactions exhibit strong spatiotemporal coupling properties. By representing each vehicle as a graph node and the corresponding interaction relationships between vehicles as edges, multi-vehicle dynamic interactions can be naturally formulated as an undirected graph structure. This graph-based modeling approach effectively captures time-varying inter-vehicle dependencies and provides a structured representation basis for subsequent driving intent recognition and trajectory prediction.

## 3. DyGNN–Transformer-Based Trajectory Prediction Model

**Figure 2:**
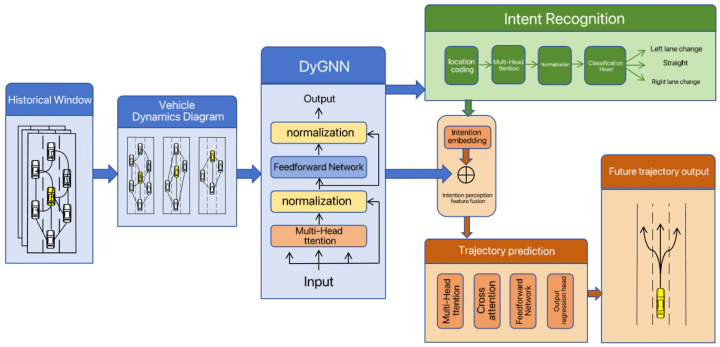
Trajectory Prediction Model.

The overall trajectory prediction framework proposed in this paper is illustrated in Figure 2 and consists of three key modules: dynamic interaction feature extraction, Transformer-based driving intent recognition, and intent-guided trajectory prediction. In contrast to conventional approaches that rely solely on historical trajectory information, the proposed framework introduces targeted enhancements in both model architecture and information flow mechanisms.

At the feature modeling stage, a Dynamic Graph Neural Network is introduced to explicitly represent spatial interaction relationships among multiple vehicles. Conventional sequence-based models typically concatenate surrounding-vehicle features as input, which makes it difficult to characterize time-varying interaction strength and influence ranges between vehicles. To address this limitation, a dynamic spatiotemporal interaction graph is constructed to provide a unified representation of complex spatial dependencies within a graph neural network framework. With this design, the model can adaptively learn the influence weights of different neighboring vehicles on the motion decisions of the target vehicle, thereby capturing complex yielding and competitive interaction patterns in high-speed traffic scenarios. By modeling spatial interaction complexity during the feature extraction stage, spatial and temporal dependencies are effectively decoupled. This yields more informative and discriminative interaction features for the subsequent Transformer-based intent recognition module, enabling it to focus on learning temporal dependency patterns.

At the intent modeling stage, a Transformer-based driving intent recognition module is introduced. Leveraging the self-attention mechanism, the Transformer is able to capture long-range temporal dependencies and evolving motion patterns in vehicle behavior. As a result, the model considers not only instantaneous position variations but also jointly integrates historical trajectories and learned interaction features to infer future motion tendencies. These tendencies are subsequently classified into discrete intent categories, such as left lane change, right lane change, and straight driving. Through this design, intent information is formulated as interpretable prior knowledge, thereby enhancing the model’s capability to understand and represent complex driving behaviors.

At the trajectory prediction stage, the inferred intent probabilities are integrated into the prediction module as prior constraints to guide the future trajectory generation process. Compared with conventional unconstrained prediction approaches, this strategy effectively mitigates prediction drift caused by accumulated uncertainty. Through the coordinated operation of the above modules, the proposed framework produces trajectory predictions that are more stable, physically plausible, and consistent with driving behavior logic in complex traffic scenarios.

### 3.1. DyGNN-Based Interaction Feature Extraction

Within the historical time horizon $t=(T-T_{p},\ldots ,T-1,T)$, the target vehicle $S_{0}$ and its six categories of key surrounding vehicles $S_{1}∼S_{6}$ are taken as the nodes in the graph to construct the corresponding local dynamic interaction graph:

(5) $$G\left(t\right)=\left(V(t),E(t)\right)$$

Here, the node set V(t) represents the state information of the target vehicle and its key surrounding vehicles at time step *t*, which can be written as

(6) $$V\left(t\right)=\left\{{\tilde{s}}_{0}{\left(t\right)}^{T},{\tilde{s}}_{1}{\left(t\right)}^{T},{\tilde{s}}_{2}{\left(t\right)}^{T},{\tilde{s}}_{3}{\left(t\right)}^{T},{\tilde{s}}_{4}{\left(t\right)}^{T},{\tilde{s}}_{5}{\left(t\right)}^{T},{\tilde{s}}_{6}{\left(t\right)}^{T}\right\}$$

where ${\tilde{s}}_{i}\left(t\right)$ denotes the relative state of the *i*-th vehicle at the corresponding relative position at time step *t*. To characterize the relative motion relationships among vehicles, the node feature matrix X(t) is constructed as the input to the graph neural network, and is expressed as

(7) $$X\left(t\right)=\left[\begin{array}{c} {\tilde{s}}_{0}{\left(t\right)}^{T}\\ {\tilde{s}}_{1}{\left(t\right)}^{T}\\ \vdots \\ {\tilde{s}}_{6}{\left(t\right)}^{T} \end{array}\right]=\left[\begin{array}{ccc} \Delta x_{0}\left(t\right) & \Delta y_{0}\left(t\right) & \Delta v_{0}\left(t\right)\\ \Delta x_{1}\left(t\right) & \Delta y_{1}\left(t\right) & \Delta v_{1}\left(t\right)\\ \vdots & \vdots & \vdots \\ \Delta x_{6}\left(t\right) & \Delta y_{6}\left(t\right) & \Delta v_{6}\left(t\right) \end{array}\right]$$

(8) $$\Delta x_{i}\left(t\right)=x_{i}\left(t\right)-x_{0}\left(t\right),\Delta y_{i}\left(t\right)=y_{i}\left(t\right)-y_{0}\left(t\right),\Delta v_{i}\left(t\right)=v_{i}\left(t\right)-v_{0}\left(t\right)$$

These variables represent the lateral position difference, longitudinal position difference, and velocity difference of the *i*-th vehicle relative to the target vehicle, respectively. A learnable graph structure is adopted to adaptively capture interaction relationships according to the relative states among vehicles, thereby enhancing the expressive capability of the graph model for complex traffic interaction behaviors.

Before graph propagation begins, the input feature matrix is taken as the initial node representation:

(9) $$H^{\left(0\right)}\left(t\right)=X\left(t\right)$$

Let $h_{i}^{\left(l\right)}\left(t\right)$ denote the feature representation of node *i* at time step *t* in the *l*-th layer. The node features are first linearly transformed to obtain the node embedding representation:

(10) $$Z_{i}^{\left(l\right)}\left(t\right)=W^{\left(l\right)}h_{i}^{\left(l\right)}\left(t\right)$$

where $W^{\left(l\right)}$ is the learnable weight matrix of the *l*-th layer. To further reflect the physical meaning of vehicle interactions in traffic scenarios, the interaction feature of node *j* relative to node *i* is defined as

(11) $$r_{ij}\left(t\right)=\left[\Delta x_{ij}\left(t\right),\Delta y_{ij}\left(t\right),\Delta v_{ij}\left(t\right)\right]$$

(12) $$\Delta x_{ij}\left(t\right)=x_{j}\left(t\right)-x_{i}\left(t\right),\Delta y_{ij}\left(t\right)=y_{j}\left(t\right)-y_{i}\left(t\right),\Delta v_{ij}\left(t\right)=v_{j}\left(t\right)-v_{i}\left(t\right)$$

which represent the lateral position difference, longitudinal position difference, and velocity difference of node *j* relative to node *i*, respectively. On this basis, the embedding representations of nodes *i* and *j*, together with the relative interaction feature, are jointly fed into the attention function to compute the unnormalized interaction score:

(13) $$e_{ij}^{\left(l\right)}\left(t\right)=\mathrm{LeakyReLU}\left(a^{(l)T}\left[Z_{i}^{\left(l\right)}\left(t\right)∥Z_{j}^{\left(l\right)}\left(t\right)∥r_{ij}\left(t\right)\right]\right)$$

where $a^{\left(l\right)}$ is the learnable attention parameter vector of the *l*-th layer, ∥ denotes the vector concatenation operation, and LeakyReLU(·) is the activation function. Then, the interaction scores of all nodes in the neighborhood of node *i* are normalized by the Softmax function to obtain the data-driven learnable neighborhood weights:

(14) $$A_{ij}^{\left(l\right)}\left(t\right)=\frac{\exp\left(e_{ij}^{\left(l\right)}\left(t\right)\right)}{\sum_{k\in N(i)}\exp\left(e_{ik}^{\left(l\right)}\left(t\right)\right)}$$

where N(i) denotes the neighborhood set of node *i*. Accordingly, $A_{ij}^{\left(l\right)}\left(t\right)$ represents the adaptive interaction influence strength of node *j* on node *i* at time step *t* during graph propagation in the *l*-th layer. It should be emphasized that the neighborhood relationship obtained by Equations (4)–(12) is no longer derived from manually specified hard-threshold rules, but is automatically learned by the model according to node states and interaction features. Therefore, it can more flexibly characterize the dynamic influence relationships among multiple vehicles in complex traffic flow.

Considering that the interaction strength between vehicles in real traffic scenarios is significantly correlated with spatial distance, a distance prior constraint is further introduced on the basis of learnable neighborhood weights to enhance the physical rationality of the model. First, the Euclidean distance between vehicle *i* and vehicle *j* at time step *t* is defined as

(15) $$d_{ij}\left(t\right)=\sqrt{{\left(x_{i}\left(t\right)-x_{j}\left(t\right)\right)}^{2}+{\left(y_{i}\left(t\right)-y_{j}\left(t\right)\right)}^{2}}$$

Then, the neighborhood weights are modulated by a distance decay function to obtain a weighted adjacency matrix incorporating physical priors:

(16) $${\tilde{A}}_{ij}^{\left(l\right)}\left(t\right)=\exp\left(-\frac{d_{ij}\left(t\right)}{\sigma }\right)\cdot A_{ij}^{\left(l\right)}\left(t\right)$$

where σ is the distance-decay scale parameter. Equation (14) ensures that vehicles with smaller spatial distances are assigned larger influence weights during graph propagation, enabling the model to preserve its data-driven learning capability while also taking into account the spatial interaction patterns in traffic scenarios.

After obtaining the adjacency weights fused with the distance prior, the node feature update process in the (l+1)-th layer can be expressed as

(17) $$h_{i}^{\left(l+1\right)}\left(t\right)=\sigma \left(\sum_{j=0}^{6}{\tilde{A}}_{ij}^{\left(l\right)}\left(t\right)W^{\left(l\right)}h_{j}^{\left(l\right)}\left(t\right)\right)$$

where σ(·) denotes the nonlinear activation function. Through this update process, the node representation not only preserves its own motion-state information, but also integrates the dynamically weighted interaction influences from different neighboring vehicles, thereby forming a more discriminative high-level interaction feature representation.

When the graph propagation reaches the final layer *L*, the final representations of all nodes at time step *t* can be obtained as

(18) $$H^{\left(L\right)}\left(t\right)={\left[h_{0}^{\left(L\right)}\left(t\right),h_{1}^{\left(L\right)}\left(t\right),\ldots ,h_{6}^{\left(L\right)}\left(t\right)\right]}^{T}$$

For subsequent temporal modeling, the high-level representation of the node corresponding to the target vehicle at time step *t* is taken as the interaction feature at that time step:

(19) $$e\left(t\right)=h_{0}^{\left(L\right)}\left(t\right)$$

Within the entire historical observation window, the interaction features extracted at each time step are arranged in chronological order to form the interaction feature sequence:

(20) $$E=\left[e\left(T-T_{p}+1\right),e\left(T-T_{p}+2\right),\ldots ,e\left(T\right)\right]$$

The resulting interaction feature sequence *E* is no longer a simple concatenation of single-vehicle states, but a structured high-level representation that integrates the dynamic interaction relationships between the target vehicle and its key surrounding vehicles. After being processed by the DyGNN module, the local traffic relationships embedded in the original historical trajectories are transformed into more discriminative spatiotemporal interaction features, thereby providing a more effective input basis for the subsequent Transformer module to perform temporal intention inference.

### 3.2. Driving Intent Recognition Module

To further improve the accuracy and stability of trajectory prediction, a driving intent recognition module is incorporated into the overall framework as prior constraint information [18]. Driving intent reflects the vehicle’s high-level motion tendency over a future time horizon and can be regarded as a semantic weighting over different candidate trajectories [19]. From a modeling perspective, driving intent recognition can be formulated as a multi-class sequence classification problem, in which the model infers future driving behavior based on historical motion states and interaction information.

According to real-world traffic scenarios, driving intent is categorized into three classes: left lane change, right lane change, and lane keeping. To address this task, a Transformer-based driving intent recognition model is adopted to effectively capture temporal dependencies and latent decision-making patterns from historical vehicle sequences.

The model input is defined as:

(21) $$I_{t}=\left[E_{t},S_{t}\right],$$

where $E_{t}$ denotes the interaction feature extracted by the dynamic graph neural network, which encodes the dynamic interaction relationships between the target vehicle and surrounding vehicles, and $S_{t}$ represents the historical motion state of the target vehicle. This paper employs feature concatenation to fuse the two, enabling the model to simultaneously retain both the target vehicle’s own motion information and the semantic information regarding interactions with surrounding vehicles. The concatenated fusion features are first projected onto a unified high-dimensional feature space via a linear mapping, thereby achieving dimensional alignment and a unified semantic representation of features from different sources. Subsequently, the mapped sequences are input into a Transformer encoder for temporal modelling, thereby fully learning key discriminative patterns and latent decision dependencies during the evolution of historical trajectories. Through this fusion strategy, the model is not only able to perceive the vehicle’s own motion trends but can also effectively utilise dynamic interaction information within complex traffic environments, thereby enhancing the accuracy and continuity of driving intention recognition. At the output stage, the intent recognition module applies a Softmax layer to normalize the output of the Transformer decoder, producing a probability distribution over the three driving intent categories. This forms an intent probability vector $P=[w_{1},w_{2},w_{3}]$, where $w_{1}$, $w_{2}$, and $w_{3}$ denote the predicted probabilities of left lane change [20], right lane change, and lane keeping, respectively. Since a vehicle exhibits only one definitive driving intent at each time step in real-world scenarios, the intent category corresponding to the maximum probability is selected as the final prediction. The selected intent is encoded as a one-hot vector, while the remaining entries are set to zero. Therefore, the output of the driving intent recognition module takes one of the following forms: [1, 0, 0], [0, 1, 0], or [0, 0, 1], corresponding to left lane change, right lane change, and lane keeping, respectively [21].

### 3.3. Trajectory Prediction Module

Based on the inferred driving intent, an intent-guided trajectory prediction module is further constructed to achieve accurate modeling and prediction of the target vehicle’s future motion trajectory. Compared with conventional prediction methods that rely solely on historical trajectory information, the incorporation of high-level driving intent as a prior constraint enables the model to explicitly distinguish trajectory evolution patterns under different driving behaviors, thereby improving prediction accuracy and stability in highly nonlinear scenarios such as lane changes. The input to the trajectory prediction module consists of three components: the historical motion state sequence of the target vehicle, the interaction feature sequence extracted by the dynamic graph neural network, and the intent feature sequence generated by the driving intent recognition module. Specifically, given the prediction time step *T* and the historical observation window length $T_{P}$, the input to the trajectory prediction module is defined as:

(22) $$Z_{t}=\left[E_{t},S_{t},I_{t}\right],\;t\in \left[T-T_{P}+1,T\right],$$

where $E_{t}$ denotes the interaction feature at time step *t*, $S_{t}$ represents the historical motion state of the target vehicle, and $I_{t}$ denotes the one-hot encoded driving intent vector obtained from the intent recognition module. In the encoding stage, the trajectory prediction module first applies a linear projection to the fused input sequence, mapping it into a unified high-dimensional feature space. Positional encoding is then added to preserve temporal order information. Subsequently, multiple Transformer encoder layers are employed to model the sequence [22], where the self-attention mechanism captures long-range dependencies across different historical time steps and integrates the global constraint effect of intent features on motion trends. In the decoding stage [23], an autoregressive strategy is adopted to generate future trajectory points step by step. Using the high-level temporal features produced by the encoder as the initial condition, the model predicts the two-dimensional position coordinates of the target vehicle at each future time step $t_{1}\in \left[T+1,T+T_{F}\right]$, defined as:

(23) $$O\left(t_{1}\right)=\left(\hat{X}\left(t_{1}\right),\hat{Y}\left(t_{1}\right)\right),$$

where $\hat{X}\left(t_{1}\right)$ and $\hat{Y}\left(t_{1}\right)$ denote the predicted longitudinal and lateral positions, respectively. The predicted output at the current time step is fed back into the decoder as input for the next time step, enabling continuous multi-step trajectory prediction.

## 4. Experimental Preparation

**Figure 3:**
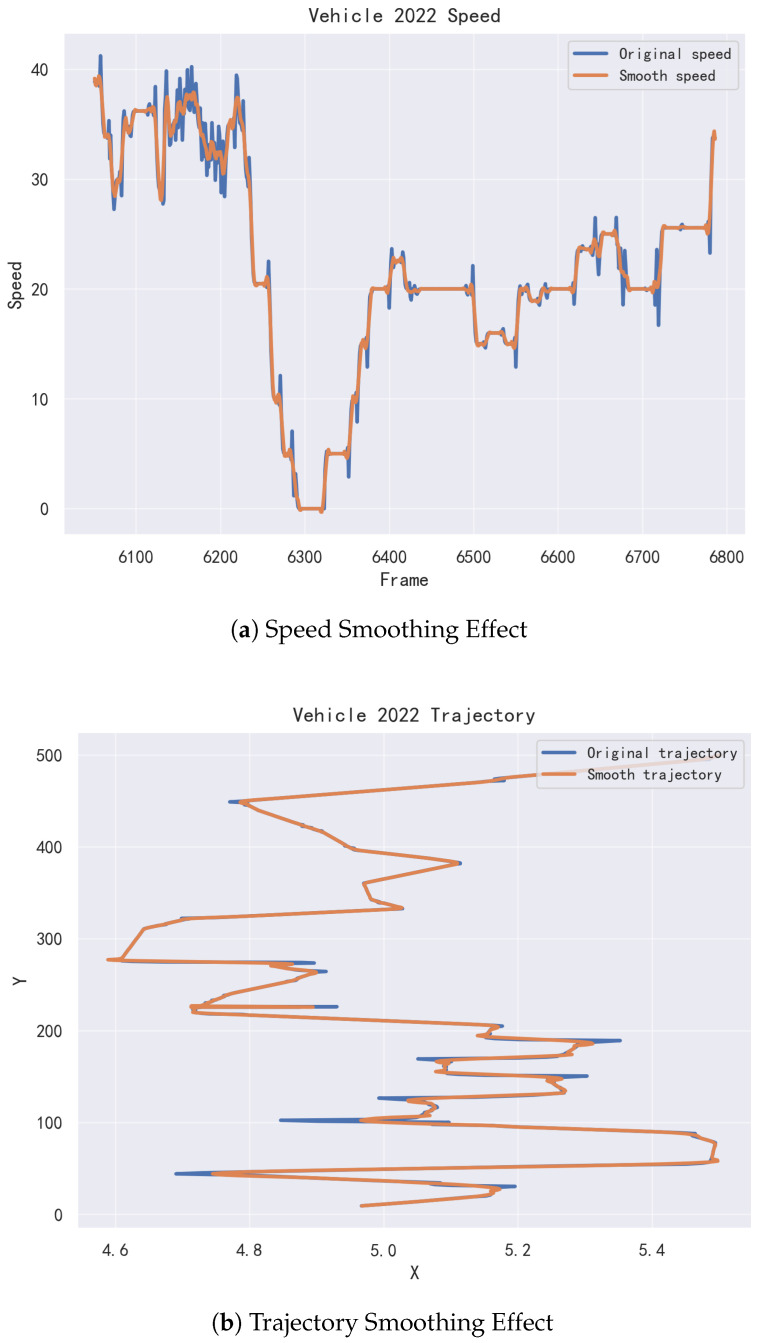
Smoothness effect comparison for Vehicle No. 2022.

This paper utilizes data from the US-101 section of the Naturalistic Driving Study (NGSIM) dataset [24] and the highD dataset for experimental validation [25]. To improve the quality of the raw vehicle trajectory data and ensure consistency with driving intent annotations, systematic preprocessing was performed on both datasets. The preprocessing procedure mainly consists of two steps: trajectory smoothing and lane-change intent continuity correction. First, to address measurement noise and local fluctuations commonly present in real-world traffic data, Savitzky–Golay filtering was applied to smooth vehicle trajectories. By performing low-order polynomial least-squares fitting within a sliding window, this method can effectively suppress high-frequency noise while preserving the local geometric characteristics of the trajectory, making it particularly suitable for continuous, smooth, yet noisy time-series data such as vehicle motion trajectories. Specifically, continuous variables including the lateral position, longitudinal position, and vehicle velocity were filtered independently. The window length was set to 11, and the polynomial order was set to 3. Taking the trajectory data of a lane-changing vehicle as an example (vehicle number 2022 is selected in the figure), the comparison between the filtered data and the original data is shown in Figure 3.

To improve the consistency and reliability of lane-change intent annotations, a motion trend-based intent completion strategy was introduced to correct frames that correspond to the early phase of lane-change maneuvers. Specifically, for time intervals preceding annotated lane-change events, several historical frames were examined, and the trend of the vehicle’s lateral coordinate was analyzed. If these frames exhibited sustained and monotonic lateral displacement consistent with the subsequent lane-change direction, the vehicle was considered to have already entered the preparation or execution phase of the lane-change maneuver. In such cases, the driving intent label of the corresponding frames was corrected from “lane keeping” to the appropriate lane-change intent. Previous studies have reported that the typical duration of a lane-change maneuver is approximately 8 s [26], while Han Hao et al. [27] demonstrated that a 3-s historical sequence provides sufficient information for trajectory prediction. Based on these findings, this study extracted trajectory segments consisting of 3 s before and 4 s after the lane-change point. The 3-s historical trajectory was annotated with driving intent labels and used for training and validation of the driving intent recognition module, while the subsequent 4-s trajectory was used for training and validation of the trajectory prediction module. To reduce the influence of class imbalance on model training and evaluation, a balanced sampling strategy was adopted for both the NGSIM and highD datasets, ensuring that the number of samples in each driving intent category remained consistent within each dataset. For the NGSIM dataset, a total of 6696 valid trajectory samples were selected, including 2232 samples for each intent category, namely left lane change, lane keeping, and right lane change. For the highD dataset, a total of 6444 valid trajectory samples were selected, including 2148 samples for each driving intent category. In both datasets, 80% of the samples were used for training and the remaining 20% were used for testing.

## 5. Model Training

### 5.1. Training of Intent Recognition Models

The cross-entropy loss function is adopted as the training objective. The cross-entropy loss for a single sample can be expressed as

(24) $$L_{ce}=-\sum_{c=1}^{3}y_{c}\log\left({\hat{y}}_{c}\right)$$

where ${\hat{y}}_{c}$ denotes the predicted probability that the sample belongs to the *c*-th driving intention class, and $y_{c}$ denotes the ground-truth label value of the corresponding class. The cross-entropy loss can effectively measure the discrepancy between the predicted distribution and the true distribution. When the model assigns a higher probability to the correct class, the loss value becomes smaller. Conversely, if the model assigns a high probability to an incorrect class, the loss increases significantly. By minimizing this loss function, the model can gradually learn the mapping relationship among historical motion states, interaction relationships, and driving intentions.

The training hyperparameters used in this study are uniformly configured, as shown in Table 1. The Adam optimizer is adopted. The initial learning rate is set to 0.001, the batch size is set to 64, the number of training epochs is set to 100, and the dropout rate is set to 0.1.

The distance-decay coefficient was set to 15 m to reflect the effective local interaction range among vehicles in highway scenarios, so that nearby vehicles maintain dominant influence during graph propagation while the effects of more distant vehicles gradually decrease with distance. If this parameter is set too small, the model may overemphasize only very close vehicles and ignore surrounding vehicles that still have practical influence; if it is set too large, the discriminative ability of the distance prior will be weakened, allowing distant vehicles to introduce more redundant interaction information. Therefore, 15 m provides a reasonable balance between local interaction sensitivity and long-range noise suppression.

### 5.2. Training of Trajectory Prediction Models

Considering that future trajectory prediction is essentially a multi-step continuous regression problem, this study constructs the loss function based on the prediction error of the two-dimensional position coordinates of the target vehicle at each future time step.

The ground-truth position of the target vehicle at future time step $t_{f}$ is defined as

(25) $$P\left(t_{f}\right)=\left(x\left(t_{f}\right),\;y\left(t_{f}\right)\right)$$

The corresponding position predicted by the model is denoted as $\hat{P}\left(t_{f}\right)$. Then, the regression loss for trajectory prediction is defined as the mean squared error (MSE) loss:

(26) $$L_{\mathrm{traj}}=\frac{1}{T_{F}}\sum_{t_{f}=T+1}^{T+T_{F}}\left[{\left(\hat{x}\left(t_{f}\right)-x\left(t_{f}\right)\right)}^{2}+{\left(\hat{y}\left(t_{f}\right)-y\left(t_{f}\right)\right)}^{2}\right]$$

The Adam optimizer is adopted to update the model parameters during training. The initial learning rate is set to 0.001, the batch size is set to 64, the number of training epochs is set to 100, and the dropout rate is set to 0.1.

To prevent overfitting during training, an Early Stopping strategy is further introduced in this study, with the patience set to 10. Specifically, when the performance on the validation set does not improve for 10 consecutive epochs, the training process is terminated and the current best model parameters are saved. The specific parameter settings are listed in Table 2.

## 6. Experiment and Analysis

### 6.1. Analysis of Driving Intent Experiment Results

To evaluate the recognition performance of the proposed DyGNN–Transformer (DGT) based model for identifying the driving intentions of surrounding vehicles, balanced sets of historical trajectory samples were constructed from the NGSIM and highD datasets. The proposed model was compared with driving intention recognition models based on LSTM, BiLSTM, GAT-BiGRU, and Transformer. Table 3 presents the comparison results on the NGSIM dataset.

As shown in Table 3, DGT achieved the best performance in driving intention recognition on the NGSIM dataset, with recognition accuracies of 90.73%, 96.54%, and 91.34% for left lane change, lane keeping, and right lane change, respectively, and an average accuracy of 92.87%. Compared with LSTM, BiLSTM, GAT-BiGRU, and Transformer, the average accuracy was improved by 6.55, 4.83, 3.75, and 2.36 percentage points, respectively. In particular, compared with the baseline Transformer, the introduction of the DyGNN module increased the average accuracy from 90.51% to 92.87%, indicating that the DyGNN module itself contributed a performance gain of 2.36 percentage points.

The comparison results of intention recognition performance based on the highD dataset are shown in Table 4.

As shown in Table 4, the driving intention recognition results of all models on the highD dataset exhibit a trend consistent with that observed on the NGSIM dataset. Among them, the proposed DGT model achieved recognition accuracies of 92.32%, 96.04%, and 91.99% for left lane change, lane keeping, and right lane change, respectively, with an average accuracy of 93.45%, outperforming all the other comparative models. Compared with the Transformer model, the proposed model improved the average accuracy by 1.89 percentage points, further demonstrating that the introduction of DyGNN into the Transformer framework can effectively enhance driving intention recognition performance. In addition, the recognition accuracy of all models for the lane-keeping intention was generally higher than that for left and right lane changes. This is mainly because lane-keeping behavior is more stable, whereas lane-changing behavior exhibits a gradual evolution before execution and is more easily influenced by the interaction with surrounding vehicles, making it relatively more difficult to recognize. Nevertheless, the DGT model still maintained high recognition accuracy for both left and right lane change categories, indicating that it can effectively capture critical interaction cues and temporal variation information before lane changes occur. Overall, the experimental results on the highD dataset further verify the effectiveness of the proposed method and demonstrate that the model shows relatively stable performance across different datasets, with good generalization ability and robustness.

**Figure 4:**
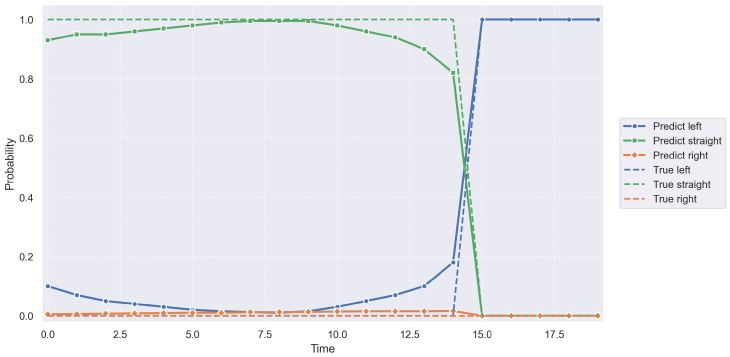
Intent Prediction Confidence.

As shown in Figure 4, the model maintains consistently high prediction confidence for the lane-keeping intent prior to the lane-change maneuver, while the probabilities of left and right lane-change intents remain low, with no evident misclassifications. As the vehicle approaches the lane-change moment, the probability of the lane-keeping intent decreases sharply, accompanied by a rapid increase in the corresponding lane-change intent probability. This transition closely aligns with the actual intent switching point. Furthermore, the predicted intent probabilities exhibit smooth and continuous transitions throughout the entire process, without noticeable oscillations or abrupt fluctuations. This indicates that the proposed model can accurately capture the temporal evolution of driving intent and effectively identify the onset of lane-change maneuvers. As a result, the inferred intent provides reliable prior information, which contributes to improving the accuracy and stability of subsequent trajectory prediction.

### 6.2. Analysis of Trajectory Prediction Experiment Results

To comprehensively evaluate trajectory prediction performance, this study adopts the Root Mean Square Error (RMSE) and Final Displacement Error (FDE) as evaluation metrics. Specifically, RMSE is used to measure the overall deviation between the predicted trajectory and the ground-truth trajectory over the entire prediction horizon, thereby reflecting the fitting accuracy of the model to future trajectories. In contrast, FDE measures the deviation between the predicted final position and the true final position, which more directly indicates the model’s ability to predict the vehicle’s final destination. Since trajectory prediction requires not only accurate fitting of the overall trajectory shape but also precise estimation of the final position, the joint use of RMSE and FDE enables a more comprehensive assessment of model performance.

The RMSE is defined as follows:

(27) $$RMSE=\sqrt{\frac{1}{T}\sum_{t=1}^{T}\left[{\left({\hat{x}}_{t}-x_{t}\right)}^{2}+{\left({\hat{y}}_{t}-y_{t}\right)}^{2}\right]}$$

The FDE is defined as follows:

(28) $$FDE=\sqrt{{\left({\hat{x}}_{T}-x_{T}\right)}^{2}+{\left({\hat{y}}_{T}-y_{T}\right)}^{2}}$$

where *T* denotes the prediction horizon length; $\left(x_{t},y_{t}\right)$ represents the ground-truth position coordinates at time step *t*; $\left({\hat{x}}_{t},{\hat{y}}_{t}\right)$ denotes the predicted position coordinates at time step *t*; and $\left(x_{T},y_{T}\right)$ and $\left({\hat{x}}_{T},{\hat{y}}_{T}\right)$ represent the ground-truth final position and the predicted final position, respectively. A smaller RMSE indicates better overall trajectory fitting performance over the prediction horizon, whereas a smaller FDE indicates higher accuracy in predicting the final position of the target vehicle.

**Figure 5:**
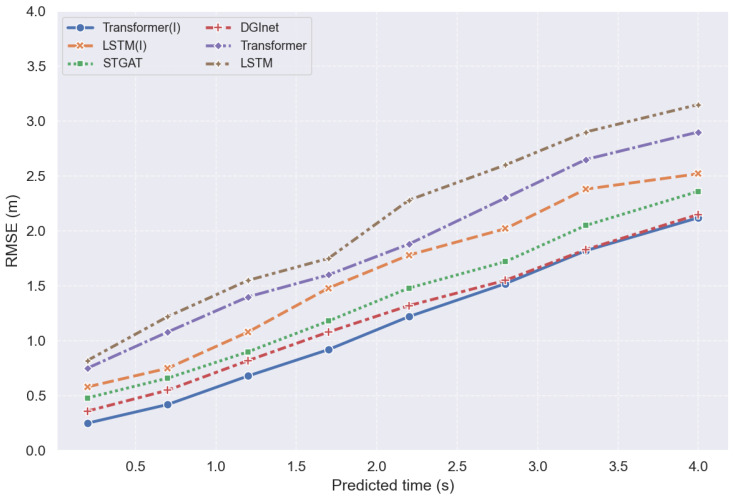
Comparison of Inference Time and RMSE.

To systematically evaluate the performance of the proposed method in vehicle trajectory prediction tasks, several baseline models were selected for comparison, including the LSTM model, LSTM(I) incorporating driving intent information [4], the Transformer model [28], the STGAT model, the DGInet model and Transformer(I) integrating dynamic graph interaction modeling and intent recognition modules. Among them, Transformer(I) denotes the trajectory prediction model proposed in this study. The LSTM model adopts a conventional encoder–decoder architecture for trajectory prediction, whereas LSTM(I) further incorporates the same driving intention prediction mechanism as that used in the proposed model, in order to investigate the contribution of intention information to the performance improvement of sequence models. The Transformer model relies only on the self-attention mechanism to model temporal features and does not include intention recognition or interaction modeling modules. The STGAT model jointly captures spatial interaction relationships among vehicles and temporal evolution characteristics by constructing a spatio-temporal graph attention mechanism, thereby effectively characterizing trajectory variation patterns in multi-vehicle interaction scenarios. The DGlnet model employs a dynamic graph interaction network to model the time-varying interaction relationships among vehicles and captures temporal dependencies in complex traffic environments through dynamic graph structure updates, thus improving trajectory prediction performance. To ensure the fairness of the comparative experiments, all models were trained and tested using the same data preprocessing procedure and input information, with vehicle motion states and surrounding vehicle interaction information being uniformly used for prediction. The trajectory prediction performance of different models is evaluated using Root Mean Square Error (RMSE), and the comparison results are presented in Table 5 and Figure 5.

Table 5 shows that the RMSE values of all models gradually increase as the prediction horizon extends, reflecting the cumulative nature of trajectory prediction errors over time. The average RMSE of LSTM is 2.31, while after introducing driving intention information, the average RMSE of LSTM(I) decreases to 1.82, indicating that intention priors can effectively reduce prediction uncertainty. STGAT and DGInet, which explicitly model vehicle interaction relationships, both achieve better performance than traditional temporal models, with DGInet showing superior performance. On this basis, the proposed Transformer(I), after incorporating the intention recognition module, further reduces the average RMSE from 2.08 for Transformer to 1.35, further demonstrating that driving intention information plays an important auxiliary role in trajectory prediction.

The results of the FDE for the different models are shown in Table 6.

As shown in Table 6, the FDE values of all models gradually increase as the prediction horizon extends, indicating that the endpoint position error accumulates over time. Unlike RMSE, which emphasizes the overall trajectory fitting accuracy, FDE better reflects the model’s ability to predict the vehicle’s final position. The results show that Transformer(I) achieves the lowest FDE at all prediction horizons, with an average value of 1.49. Compared with LSTM, Transformer, LSTM(I), STGAT, and DGInet, the average FDE of Transformer(I) is reduced by 1.14, 0.84, 0.56, 0.39, and 0.14, respectively, indicating that after incorporating the intention recognition module, the model not only improves the overall trajectory fitting performance but also more accurately captures the vehicle’s future motion direction and final position variation trend.

**Figure 6:**
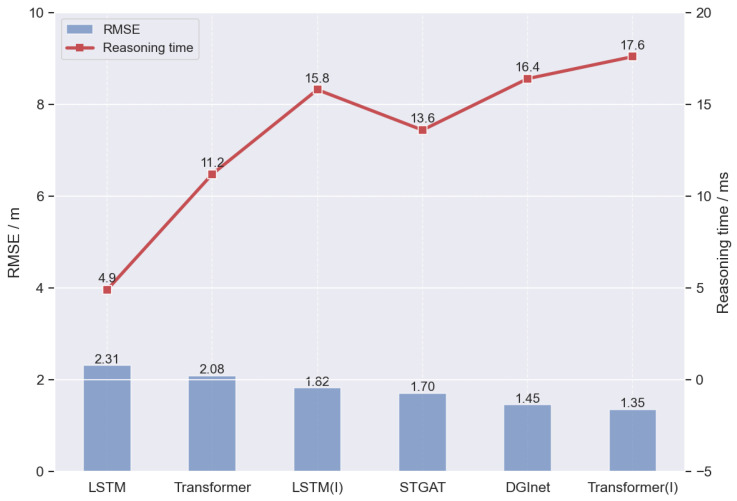
Comparison of Inference Time and RMSE.

As shown in Figure 6, the compared models exhibit significant differences in prediction accuracy and inference efficiency.

LSTM achieves the shortest inference time of 4.9 ms, but its RMSE reaches 2.31, indicating that although the conventional recurrent structure is computationally efficient, its prediction accuracy is relatively limited. Transformer increases the inference time to 11.2 ms while reducing the RMSE to 2.08, suggesting that the self-attention mechanism can improve long-range temporal modeling to some extent. After introducing driving intention information, LSTM(I) further reduces the RMSE to 1.82, but its inference time rises to 15.8 ms, confirming that intention priors can effectively constrain future trajectory evolution. STGAT and DGInet, which explicitly model inter-vehicle interactions, achieve lower RMSE values of 1.70 and 1.45, respectively, with inference times of 13.6 ms and 16.4 ms, showing that interaction modeling, especially dynamic graph interaction modeling, can further improve prediction performance at the cost of additional computation. Among all models, the proposed Transformer(I) achieves the best overall result, with the lowest RMSE of 1.35 and an inference time of 17.6 ms. Although its computational cost is the highest, it remains within the millisecond level and therefore still satisfies real-time requirements to a certain extent.

**Figure 7:**
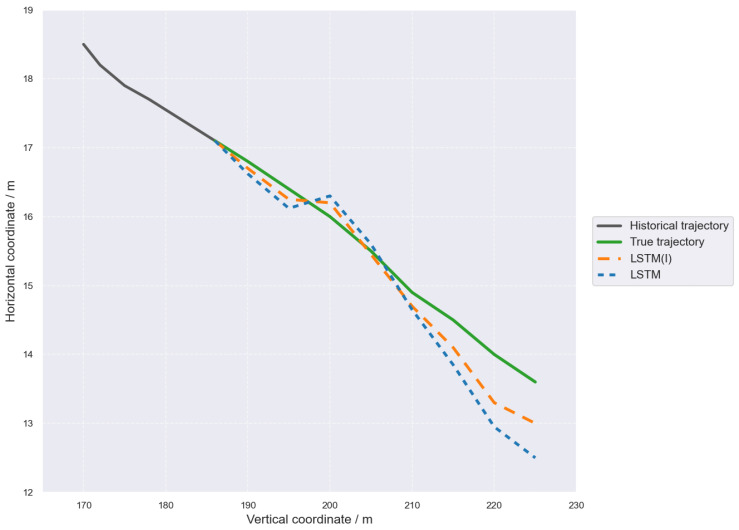
Comparison of Predicted Trajectories by LSTM and LSTM(I).

The vehicle trajectory and position predictions for LSTM and LSTM(I) are shown in Figure 7. Compared with the LSTM model that predicts trajectories solely based on historical temporal features, the LSTM(I) model incorporating driving intention information generates predicted trajectories that are closer to the ground-truth trajectory, especially showing better tracking capability in the middle and later stages of the prediction horizon.

**Figure 8:**
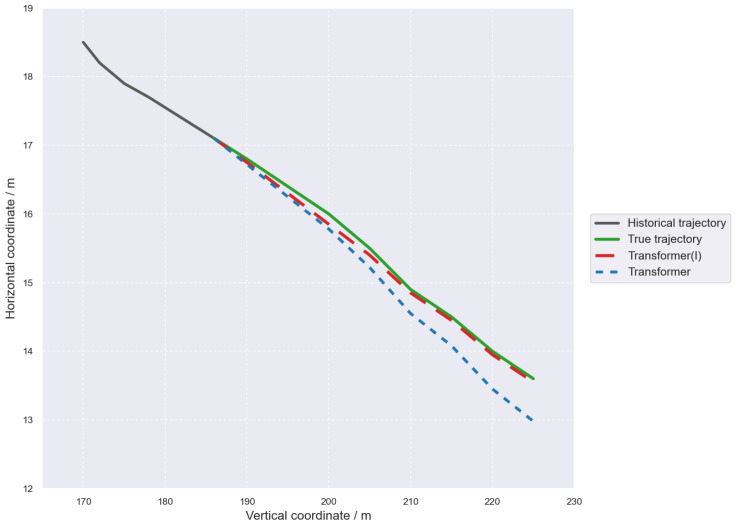
Comparison of Predicted Trajectories by Transformer and Transformer(I).

The vehicle trajectory and position predictions for Transformer and Transformer(I) are shown in Figure 8.

As can be seen from the figure, both the Transformer and Transformer(I) models are able to capture the overall variation trend of the target vehicle’s future trajectory, but there are obvious differences in prediction accuracy between them. Compared with the Transformer model, which only models the historical trajectory sequence, the Transformer(I) model incorporating driving intention information produces predicted trajectories that are closer to the ground-truth trajectory, especially showing better fitting performance in the middle and later stages of the prediction horizon.

**Figure 9:**
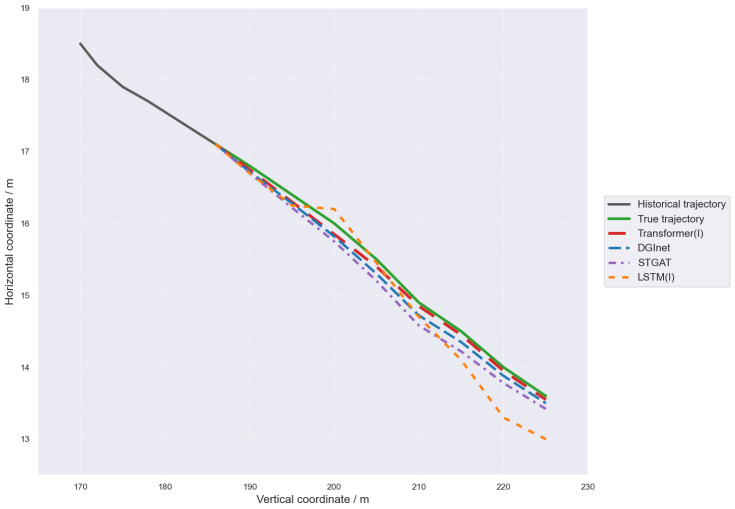
Comparison of Trajectories Predicted by Different Models.

As shown in Figure 9, all models are able to predict the overall variation trend of the target vehicle’s future trajectory to a certain extent, but there are obvious differences in trajectory fitting accuracy and late-stage prediction performance. Overall, the proposed Transformer(I) model generates predicted trajectories that are closest to the ground-truth trajectory and maintains a high degree of consistency throughout the entire prediction horizon, indicating that it can more accurately characterize the future motion trend of the vehicle. In contrast, although DGInet and STGAT can also reasonably reflect the trajectory variation direction, they still show certain deviations in the middle and later stages of prediction. LSTM(I), however, exhibits a more obvious deviation in the later stage, with a relatively larger endpoint position error. These results further demonstrate that, after incorporating the intention module, the model can effectively improve the accuracy and stability of future trajectory prediction.

## 7. Conclusions

This paper addresses the challenge of predicting vehicle trajectories in complex traffic scenarios by proposing a trajectory prediction method that integrates dynamic graph neural networks with driving intent recognition. By explicitly modeling dynamic inter-vehicle interactions and introducing a motion trend-based intent completion strategy, the proposed approach improves the temporal continuity and consistency of intent inference. Furthermore, incorporating driving intent as a prior constraint into the trajectory prediction process effectively reduces prediction uncertainty and enhances trajectory estimation accuracy. Extensive experimental results on real-world traffic datasets demonstrate that the proposed method consistently outperforms baseline models across multiple prediction horizons. In particular, it achieves superior accuracy, stability, and robustness in complex interactive scenarios such as lane-change maneuvers. These improvements highlight the effectiveness of jointly modeling interaction dynamics and behavioral intent for trajectory prediction. Future work will further explore the modeling of interactions among more complex traffic participants and scenarios involving the parallel evolution of multiple intentions, thereby enhancing the model’s applicability in real-world traffic environments.

## Figures and Tables

**Table 1:** Main parameter settings of each model.

Module	Parameter	Value
DyGNN	Number of graph neural network layers	2
	Node input feature dimension	3
	Hidden feature dimension	64
	Number of graph attention heads	4
	Distance decay coefficient	15 m
Transformer	Number of encoder layers	4
	Model feature dimension	128
	Number of multi-head attention heads	4
	Hidden dimension of feed-forward network	256

**Table 2:** Main parameter settings of the model.

Module	Parameter	Value
Transformer	Number of encoder layers	4
	Model feature dimension	128
	Number of multi-head attention heads	4
	Hidden dimension of feed-forward network	256

**Table 3:** Comparison of Intention Recognition Performance on the NGSIM Dataset.

Model	Driving Intent	Samples	Accuracy (%)	Avg. Acc. (%)
LSTM	Left Change	2232	85.74	86.32
	Lane Keeping	2232	87.62	
	Right Change	2232	85.61	
BiLSTM	Left Change	2232	87.26	88.04
	Lane Keeping	2232	89.84	
	Right Change	2232	87.03	
GAT-BiGRU	Left Change	2232	88.15	89.12
	Lane Keeping	2232	90.42	
	Right Change	2232	88.79	
Transformer	Left Change	2232	88.69	90.51
	Lane Keeping	2232	92.13	
	Right Change	2232	90.70	
DGT	Left Change	2232	90.73	92.87
	Lane Keeping	2232	96.54	
	Right Change	2232	91.34	

**Table 4:** Comparison of Intention Recognition Performance on the highD Dataset.

Model	Driving Intent	Samples	Accuracy (%)	Avg. Acc. (%)
LSTM	Left Change	2148	87.06	87.74
	Lane Keeping	2148	89.39	
	Right Change	2148	86.76	
BiLSTM	Left Change	2148	88.59	89.26
	Lane Keeping	2148	90.97	
	Right Change	2148	88.22	
GAT-BiGRU	Left Change	2148	89.48	90.16
	Lane Keeping	2148	91.82	
	Right Change	2148	89.18	
Transformer	Left Change	2148	90.18	91.56
	Lane Keeping	2148	93.36	
	Right Change	2148	91.13	
DGT	Left Change	2148	92.32	93.45
	Lane Keeping	2148	96.04	
	Right Change	2148	91.99	

**Table 5:** Comparison of trajectory prediction RMSE among different models.

Horizon (s)	LSTM	Transformer	LSTM(I)	STGAT	DGInet	Transformer(I)
1	1.42	1.25	0.93	0.88	0.68	0.55
2	2.01	1.73	1.61	1.56	1.24	1.05
3	2.69	2.44	2.18	2.01	1.72	1.69
4	3.13	2.89	2.54	2.36	2.15	2.12
Average	2.31	2.08	1.82	1.70	1.45	1.35

**Table 6:** Comparison of trajectory prediction FDE among different models.

Horizon (s)	LSTM	Transformer	LSTM(I)	STGAT	DGInet	Transformer(I)
1	1.56	1.34	1.02	0.94	0.76	0.63
2	2.26	1.95	1.78	1.63	1.38	1.18
3	3.02	2.71	2.43	2.24	1.96	1.88
4	3.68	3.31	2.96	2.71	2.43	2.28
Average	2.63	2.33	2.05	1.88	1.63	1.49

## Data Availability

The data presented in this study are available from the corresponding author upon request, provided that permission is granted by the authorized personnel.
